# Development and Validation of Novel PCR Assays for the Diagnosis of Bovine Stephanofilariasis and Detection of *Stephanofilaria* sp. Nematodes in Vector Flies

**DOI:** 10.3390/pathogens10091211

**Published:** 2021-09-17

**Authors:** Muhammad Noman Naseem, Ali Raza, Rachel Allavena, Michael McGowan, Jess A. T. Morgan, Constantin Constantinoiu, Ala E. Tabor, Peter James

**Affiliations:** 1 Queensland Alliance for Agriculture and Food Innovation, Centre for Animal Science, The University of Queensland, St. Lucia 4072, Australia; m.naseem@uqconnect.edu.au (M.N.N.); a.raza@uq.edu.au (A.R.); a.tabor@uq.edu.au (A.E.T.); 2 School of Veterinary Science, The University of Queensland, Gatton 4343, Australia; r.allavena@uq.edu.au (R.A.); m.mcgowan@uq.edu.au (M.M.); 3 Department of Agriculture and Fisheries, Queensland Government, Brisbane 4102, Australia; Jessica.Morgan@daf.qld.gov.au; 4 College of Public Health, Medical & Veterinary Sciences, James Cook University, Townsville 4811, Australia; constantin.constantinoiu@jcu.edu.au; 5 School of Chemistry & Molecular Biosciences, The University of Queensland, St. Lucia 4072, Australia

**Keywords:** *Stephanofilaria*, buffalo fly lesion, ITS2, *cox1*, cattle dermatitis

## Abstract

Background: *Stephanofilaria* spp. nematodes are associated with cutaneous lesions in cattle and other livestock and mammalian wildlife species. In Australia, *Haematobia irritans exigua,* commonly known as buffalo fly (BF) transmits a well-described but presently unnamed species of *Stephanofilaria*, which has been speculatively implicated in the aetiology of BF lesions. The sensitivity of current techniques for detecting *Stephanofilaria* spp. in skin lesions and vector species is low, and there is no genomic sequence for any member of the genus *Stephanofilaria* currently available in sequence databases. Methods: To develop molecular assays for the detection of the Australian *Stephanofilaria* sp., skin biopsies were collected from freshly slaughtered cattle with typical lesions near the medial canthus. Adult nematodes and microfilariae were isolated from the biopsies using a saline recovery technique. The nematodes were morphologically identified as *Stephanofilaria* sp. by scanning electron microscopy. DNA was extracted and the internal transcribed spacer 2 (ITS2) region of rDNA, and the cytochrome c oxidase subunit 1 (*cox1*) region of mtDNA was amplified and sequenced. *Stephanofilaria* sp. specific polymerase chain reaction (PCR) and qPCR assays (SYBR Green^®^ and TaqMan™) were developed and optimised from the novel ITS2 sequence obtained. The specificity of each assay was confirmed by testing against nematode species *Onchocerca gibsoni* and *Dirofilaria immitis*, as well as host (bovine) and BF DNA. Results: Scanning electron microscopy of the anterior and posterior ends of isolated nematodes confirmed *Stephanofilaria* sp. A phylogenetic analysis of the *cox1* sequence demonstrated that this species is most closely related to *Thelazia callipaeda*, a parasitic nematode that is a common cause of thelaziasis (or eyeworm infestation) in humans, dogs, and cats. Both conventional and qPCR assays specifically amplified DNA from *Stephanofilaria* sp. Conventional PCR, TaqMan™, and SYBR Green^®^ assays were shown to detect 1 ng, 1 pg, and 100 fg of *Stephanofilaria* DNA, respectively. Both qPCR assays detected DNA from single *Stephanofilaria* microfilaria. Conclusion: Molecular diagnostic assays developed in this study showed high specificity and sensitivity for *Stephanofilaria* sp. DNA. The availability of an accurate and sensitive PCR assay for *Stephanofilaria* will assist in determining its role in the pathogenesis of cattle skin lesions, as well as in understanding its epidemiological dynamics. This assay may also have application for use in epidemiological studies with other species of *Stephanofilaria*, most particularly closely related *S. stilesi*, but this will require confirmation.

## 1. Introduction

Filarioid nematodes are a group of tissue-dwelling nematodes of vertebrates, transmitted by hematophagous arthropods. *Stephanofilaria* spp. are small (up to 8 mm) parasitic roundworms, causing cutaneous lesions mainly in cattle, but have also been reported to cause dermatitis in other livestock and mammalian wildlife species including buffaloes, goats, pigs, rhinoceroses, hippopotami, and giraffes [1,2,3,4,5,6,7]. Although the systematics of this group is somewhat fluid, a recent placement of the genus *Stephanofilaria* in the Nematoda is order Spirurida, superfamily Filariodidea, family Filarioidea [8]. *Stephanofilaria stilesi*, *S. assamensis*, *S. dedoesi*, *S. kaeli* and *S. okinawaensis* have been reported in cattle skin lesions from the United States, Russia, India, Indonesia, Malaysia, and Japan, respectively (reviewed by Johnson [6]). 

In Australia, cattle are infested by an unnamed species of *Stephanofilaria*. This species is found widely in cattle in northern Australia in association with the buffalo fly, *Haematobia irritans exigua* (BF). Johnson [6] has provided a full description of the Australian *Stephanofilaria* sp. complete with detailed morphological diagrams, electron microscope images, and a tentative key to species, including the Australian type. He differentiated the Australian *Stephanofilaria* from *S. stilesi* on the basis of the cephalic armature, with 4–6 cephalic spines present in *S. stilesi* but absent in the Australian type. However, he did not elevate the Australian type to species status because of difficulty in obtaining samples of *S. stilesi* for detailed comparison. 

In cattle, adult nematodes live in the dermis forming cysts at the base of hair follicles and release membrane-encapsulated microfilariae into the dermal tissues. Microfilariae of *S. stilesi* and *Stephanofilaria* sp. in Australia are surrounded by a vitelline membrane (up to 60 μm diameter) and are thought to be the stage ingested by vector flies [6,7]. The microfilaria develops through the first and second larval stages (L1 and L2) to infective larva (L3) (2–3 weeks) in the intermediate host fly, before being released back into cattle skin during fly feeding [6,7]. Further information on the morphology of intermediate stages and epidemiology and pathogenesis of lesions is given by Johnson et al. [9], Johnson and Toleman [10], Shaw and Sutherland [11], and Sutherst et al. [12], respectively. Horn flies *Haematobia irritans irritans* (HF), BF and *Musca conducens* have been reported as the main biological vectors for different species of the genus *Stephanofilaria*.

In Australia, *Stephanofilaria* sp. is transmitted by BF; therefore, lesions are recognised as BF lesions [9,13], which are clinically manifested as dermatitis or wounds on the medial canthus of the eye, neck, dewlap, and ventral midline of cattle. This differs from *S. stilesi* in which lesions are found on the abdominal midline, sometimes extending to the udder [14] in cows and scrotum of bulls [15]. The lesions appear as areas with serous exudation, scab encrustation, ulceration, or circumscribed papules of 1–10 cm in diameter. Chronicity of the lesions leads to alopecia, acanthosis, and hyperkeratosis [6,9]. Development of lesions results in permanent hide damage decreasing the value of the hide and may reduce the market value of the affected stock [12]. Moreover, the presence of lesions is also a significant welfare issue and can increase the susceptibility of cattle to other infections. 

Direct visualisation of nematodes in skin sections by microscopic examination or the use of a saline recovery technique [6] are the only currently available methods for the detection of *Stephanofilaria* spp. in skin lesions. A comparison of techniques to diagnose *Stephanofilaria* sp. found 12% of lesion biopsies positive for filarial nematodes by the saline recovery technique and 32% by histopathological examination of tissue sections, whereas 10% of the samples that yielded nematodes with the saline recovery technique were found negative by histology [13]. In contrast, Miyakawa et al. [14] observed no adult *S. stilesi* or microfilariae in histopathological sections of skin lesions, but the same specimens were found to be 100% positive for nematodes using the saline recovery technique. Similarly, detection of *Stephanofilaria* sp. in the BF vector is difficult. It involves the dissection of freshly collected flies and the examination of internal contents using microscopy [6,11]. These methods are time consuming, laborious, and require training and microscopic expertise. Detection of microfilaria is particularly difficult due to their small size. Currently available diagnostic techniques for *Stephanofilaria* sp. in skin lesions and BFs are less sensitive and are laborious and imprecise. Therefore, an accurate, sensitive, and rapid diagnostic method was required.

Many studies have confirmed that PCR-based diagnostic approaches using specific primers can provide sensitive detection and identification of nematodes and their developmental stages [16,17]. Assays based on the amplification of internal transcribed spacer regions (ITS) of rDNA have been particularly useful for the detection of nematodes because there is relatively low variation in the ITS sequence within a species but large differences between species, as reported in other members of the Strongylida [18]. Several studies of PCR-based detection of helminths have been undertaken for the orders Strongylida and Ascaridida [18,19], and methods have been published for nematode species in the order Spirurida including *Dirofilaria* and *Dipetalonema* [20], *Gnathostoma* [21], *Onchocerca* and *Mansonella* [16], *Thelazia* [22], *Habronema* [23], and *Parafilaria* [24]. 

The sensitivity of molecular techniques for detecting a small quantity of nematode DNA, regardless of the developmental stage, suggested that similar approaches would be effective for the detection of *Stephanofilaria* sp. Furthermore, it is necessary to accurately detect and identify *Stephanofilaria* sp., to study its systematics, distribution, and epidemiology, and also to reliably determine the aetiology of skin lesions to design better treatment and control methods. However, no reports using PCR-based diagnosis for any species from the genus *Stephanofilaria* have been previously published, and no genome sequence information is currently available for this genus in sequence databases. In this study, we determined the *cox1* and ITS2 sequences of *Stephanofilaria* sp. and constructed a phylogeny of the *Stephanofilaria* sp. *cox1* sequence with other closely related nematode genera. In addition, molecular diagnostic assays were developed and optimised using an ITS2 based conventional PCR and qPCR methods for *Stephanofilaria* sp. detection in cattle skin lesions and the BF vector, respectively. 

## 2. Material and Methods

### 2.1. Sample Collection

Cattle skin biopsies were collected from an abattoir in Townsville (JBS Australia, Stuart, Queensland, 4811, −19.329863, 146.855438) during two different visits. All biopsies were collected from hides of freshly slaughtered cattle with obvious lesions near the medial canthus of the eyes. Grossly, lesions sampled were raised, alopecic areas of various sizes covered with dry serous crust or scab. In May 2019, biopsies were collected from 10 hides (Labelled as C-1 to C-10), and in September 2019, biopsies were collected from 15 hides (Labelled as C-11 to C-25). All biopsies were collected using 8 mm skin punches (Paramount Surgimed Ltd., New Delhi, India). From each hide, two biopsies were collected, one in normal saline solution and one in absolute ethanol for examination by the saline recovery technique and DNA extraction, respectively. 

### 2.2. Isolation of Nematode and Microfilariae

Each saline solution biopsy was cut into 5–7 slices (1–2 mm thick) using a scalpel blade (size 24, Livingstone, India) on the day of sample collection. The sliced samples were transferred to Petri plates with saline solution (0.9% sodium chloride) and incubated at room temperature overnight (method modified from Johnson [6]). Each Petri plate was inspected for nematode recovery by two observers under a stereomicroscope (SZX 10, Olympus, Shinjiku, Japan) at 2.4X magnification. 

After recovering adult nematodes, the saline solution from each Petri dish was transferred to a 15 mL screw-capped centrifugation tube using disposable plastic transfer pipettes. These tubes were centrifuged (Eppendorf 5702P) at 1500× *g* for 5 min at 20 °C. The supernatant was discarded, and the sediments were vortexed (VM1 Ratek, VIC, Australia) for 10 s. One drop from the sediments was placed onto a glass slide and covered with a glass coverslip before microscopy at 10X (Axioskop 40, Zeiss, Jena, Germany) for identification of microfilariae. Sediments found positive for microfilariae were preserved in absolute ethanol at −20 °C.

### 2.3. Morphological Examination by Scanning Electron Microscopy (SEM)

Adult nematodes (*n* = 10) isolated by saline recovery were morphologically identified using scanning electron microscopy (SEM) at the Centre for Microscopy and Microanalysis, The University of Queensland. Specimens were fixed with 4% osmium, followed by two washes with absolute ethanol to remove the osmium. Thereafter, they were dehydrated by ascending grades (25%, 50%, 75%, and 100%) of hexamethyldisilazane. After critical drying, nematodes were mounted on a carbon stub, coated with gold, and observed under the SEM (Hitachi SU3500, Osaki, Japan). All the SEM procedures were undertaken at The University of Queensland, Centre for Microscopy and Microanalysis facility. 

### 2.4. Nematode DNA Extraction

Two adult nematodes were washed twice with 500 μL of Milli-Q^®^ water in a 1.5 mL tube to remove preserving ethanol. DNA was extracted using a Qiagen DNeasy Blood and Tissue Extraction Kit as described by the manufacturer (QIAGEN Pty Ltd., Hilden, Germany, cat. Nos. 69504 & 69506). Briefly, the worms were homogenised in lysis buffer by 4 freeze cycles (45 s each) in liquid N_2_ and thawed at 50 °C using a water bath, followed by lysis in a tissue lyser (TissueLyser II, QIAGEN Pty Ltd., Hilden, Germany) with 0.5 mm zirconia beads for 30 Hz/second for 2 min. The remainder of the extraction protocol was as recommended by the manufacturer, with the exception that all extractions were eluted in 30 μL elution buffer (AE buffer). DNA concentration was measured using a NanoDrop spectrophotometer (NanoDrop 2000, Thermofisher Scientific Pty Ltd., Waltham, MA, USA).

### 2.5. Primer Design, cox1 Amplification, and Sequencing 

To amplify the *cox1* gene of mtDNA of *Stephanofilaria* sp., mtDNA sequences for *Onchocerca volvulus* (accession no. AP017695), *Loa loa* (accession no. NC016199)*, Dirofilaria immitis* (accession no. AJ537512)*,* and *Parafilaria bovicola* (accession no. MG983751) were aligned using Geneious software (version 11.1.4, Biomatters Ltd., Auckland, New Zealand). Primers were designed from conserved sequence regions targeting the desired mtDNA product. The *cox1* region of mtDNA was amplified using St_Co1_F and St_Co1_R primers. Primer pair details are described in Table 1.

PCR was performed in a total volume of 10 μL containing 1 μL of 10X *Taq* buffer, 200 μM of each dNTP, 1.5 mM of MgCl_2,_ 5 μM of each primer, 0.5 U of *Taq* DNA polymerase (QIAGEN Pty Ltd., Hilden, Germany, cat. no. 201203) and 3 ng of DNA template. The cycling conditions included an initial denaturation at 95 °C for 2 min, initial primer annealing at 50 °C for 45 s and *Taq* polymerase activation at 72 °C for 90 s, followed by 30 cycles of denaturation, annealing, and extension at 95 °C for 30 s, 50 °C for 30 s, and 72 °C for 90 s, respectively, with a final extension at 72 °C for 7 min. The amplification reaction was conducted in a T100^TM^ thermal cycler (Bio-Rad, CALIF, Hercules, CA, USA). The PCR products were run on 1% agarose gels containing gel red dye (Biotium, Fremont, CA, USA, cat. no. 41003) with 1X TBE buffer (89 mM Tris, 89 mM boric acid, 2 mM EDTA pH 8) for 40 min at 110V using a 1 kb DNA gene ruler ladder (Thermofisher Scientific, Waltham, MA, USA, cat. no. SM0311). Distinct bands were dissected under UV light, and the PCR products were purified using a PureLink^TM^ Quick gel extraction kit (QIAGEN Pty Ltd., Hilden, Germany, cat. no. K210012). Purified PCR products were sequenced through the Australian Genomic Research Facility (AGRF), The University of Queensland (St. Lucia, 4072, Queensland, Australia).

### 2.6. cox1 Based Phylogenetic Analysis

All forward and reverse sequences for *cox1* were aligned using Geneious software (v11.1.4). Endreads and other obvious errors were corrected following inspection of the DNA sequence electropherograms. The trimmed and edited DNA sequences were used to detect similarities with other available sequences in GenBank using BLASTn (https://blast.ncbi.nlm.nih.gov/Blast.cgi (accessed on 26 October 2020)). A phylogenetic tree based on *cox1* sequences was constructed using maximum likelihood analysis and a Tamura-Nei model of evolution [25] in Mega X [26]. Initial trees for the heuristic search were obtained automatically by applying Neighbor-Join and BioNJ algorithms to a matrix of pairwise distances estimated using the Tamura-Nei model and then selecting the topology with a superior log-likelihood value. The analysis involved *cox1* nucleotide sequences from eight other closely related filarial nematodes (Table 2). There was a total of 433 nucleotide positions in the final dataset without gaps. The tree was rooted using *Onchocerca* spp. as an outgroup. The robustness of the topology was tested with 1000 bootstraps [27] using the same program.

### 2.7. Development and Optimisation of Stephanofilaria sp. Specific Genetic Assays Based on ITS2 rDNA

#### Amplification and Sequencing of rDNA Segment

To amplify the rDNA segment (including partial 5.8S, complete ITS2, and partial 28S) of *Stephanofilaria* sp., rDNA sequences for *O. volvulus* (accession no. AF228576), *M. ozzardi* (accession no. MN432519), *D. repens* (accession no. KP760376) and *P. bovicola* (accession no. MG983750) were aligned using Geneious (version 11.1.4). Primers were designed within regions with consensus sequences targeting the desired rDNA products. The rDNA segment was amplified using a forward primer (St_5.8S_29F) located at the 3′ end of 5.8S rDNA and a reverse primer (28S_400R) located at the 5′ end of the 28S rDNA (Table 1). Amplification and sequencing of rDNA were performed using the protocol as described above for *cox1*.

All forward and reverse sequences for rDNA were aligned using Geneious (v11.1.4). Endreads and other obvious errors were corrected following inspection of the DNA sequence electropherograms. The trimmed and edited DNA sequences were used to detect similarities with other available sequences in GenBank using BLASTn (https://blast.ncbi.nlm.nih.gov/Blast.cgi (accessed on 26 October 2020)).

### 2.8. Development and Optimisation of a Stephanofilaria sp. Specific Conventional PCR

A *Stephanofilaria* sp. specific primer set consisting of S_ITS2_F2 and S_ITS2_R2 was designed to amplify a 199 bp product from the ITS2 region using Primer 3 (version 4.1.0). The optimum annealing temperature for *Stephanofilaria* sp. specific primers was determined by setting up a gradient PCR. The PCR reaction was set up in a total volume of 20 μL containing 10 µL of 2X Phusion Hot Start II High-Fidelity PCR Master Mix (Thermo Scientific, Waltham, MA, USA, cat. no. F-565S), 10 μM of each primer, and 3 ng of DNA template. The cycling conditions included an initial denaturation at 98 °C for 30 s, followed by 30 cycles of denaturation, annealing and extension at 98 °C for 10 s, 58 ° C for 30 s and 72 °C for 30 s, respectively, and a final extension at 72 °C for 10 min. The reaction was conducted in an Eppendorf Mastercycler^®^ pro thermal cycler (Eppendorf AG 22331 Hamburg, Germany). Gel electrophoresis was performed to ensure PCR product specificity, followed by sequencing using forward and reverse primers as described above. 

### 2.9. Development and Optimisation of SYBR Green Quantitative (q) PCR Assay

The primer set used in conventional PCR was also used for the SYBR Green-based qPCR assay. Briefly, the SYBR Green assay was conducted in a CFX96^TM^ Real-time Detection System (Bio-Rad C1000 Touch Thermal Cycler, CALIF, Hercules, CA, USA). The reaction was set up in a total volume of 20 μL containing 10 μL of PowerUP^TM^ SYBR Green Master Mix (Catalogue no. A25742, Thermofisher Scientific Pty, Waltham, MA, USA), 10 μM of each primer, and 1.5 ng of DNA template. The cycling conditions involved initial UDG activation at 50 °C for 2 min, Dual-lock ^TM^ DNA polymerase activation at 95 °C for 2 min, followed by 40 cycles of denaturation, annealing, and extension at 95 °C for 15 s, 58 °C for 15 s, and 72 °C for 60 s, respectively. Quantitation cycle (Cq) scores, corresponding to the cycle number at which the amplification curve intersects the threshold at 200 relative fluorescence units (RFU), were recorded for each sample. The instrument was set to perform the default melt curve analysis as described by the manufacturer. Every reaction was performed in duplicate with a dual negative control (no template control) in each qPCR run. 

### 2.10. Development and Optimisation of TaqMan qPCR Assay

*Stephanofilaria* sp. specific primers (forward AP326KJ_F and reverse AP326KJ_R) and a fluorescence-labelled (FAM-labelled) probe (AP326KJ_Probe) were designed from selected ITS2 sequences using the Thermofisher Custom TaqMan™ Assay designing tool (Table 1). The assay was ordered through Applied Biosystems (Thermofisher Scientific Pty, Waltham, MA, USA). This reaction was also conducted in a CFX96^TM^ Real-Time Detection System (Bio-Rad C1000 Touch Thermal Cycler, Hercules, CA, USA). The reaction was set up in a total volume of 20 µL containing 10 µL of TaqMan^TM^ Fast advance Master Mix (Catalogue no. 4444556, Thermofisher Scientific Pty, Waltham, MA, USA), 1 µL of 20X TaqMan assay mixture, and 1.5 ng of DNA template. The cycling conditions involved initial UNG activation at 50 °C for 2 min, AmpliTaq ^TM^ Fast DNA polymerase activation at 95 °C for 2 min, followed by 40 cycles of denaturation, annealing/extension at 95 °C for 3 s and 60 °C for 30 s, respectively. Quantitation cycle (Cq) scores, corresponding to the cycle number at which the amplification curve intersects the threshold line at 1000 relative fluorescence units (RFU), were recorded for each sample. Every reaction was performed in duplicate with a dual negative control (no template control) in each TaqMan qPCR run.

### 2.11. Specificity and Sensitivity Testing

The ability of the three molecular assays to specifically amplify *Stephanofilaria* sp. DNA was tested against *O. gibsoni* and *D. immitis* DNA, as both species are closely related to *Stephanofilaria*. *Onchocerca gibsoni* and *D. immitis* DNA was acquired from the Nematode Functional Genomics Laboratory (La Trobe University, Bundoora, Victoria, Australia) and College of Public Health, Medical and Veterinary Sciences (James Cook University, Townsville, Australia), respectively. The specificity of both the conventional and qPCR assays was tested by using 2.5 ng of each DNA template. 

Assay specificity was also tested against the BF vector and bovine host DNA. Laboratory reared BF (negative for *Stephanofilaria* sp.) kept under control conditions, were obtained from the EcoScience Precinct (Dutton Park 4102, Queensland, Australia). Skin biopsies were taken from cattle with no clinical *Stephanofilaria* lesions kept at the University of Queensland Pinjarra Hills research farm (UQ Animal Ethics Approval No. QAAFI469/l 8). DNA was extracted from individual BF and a cow skin biopsy using the QIAGEN DNeasy Blood and Tissue extraction kit (QIAGEN). Specificity against bovine host and fly vector DNA was tested using 2.5 ng of each DNA template in conventional and qPCRs as described above.

For sensitivity testing, DNA was extracted from 15 pooled worms using the protocol detailed above (nematode DNA extraction section). DNA was diluted 10-fold from 10 ng/µL to 1 fg/µL to determine the sensitivity of each assay.

### 2.12. Validation of Assays to Detect Microfilariae

To test the validity of the molecular assays for detecting DNA from microfilariae of *Stephanofilaria* sp., microfilariae were isolated from sediments following the saline recovery technique. DNA was extracted from single, two, and three microfilariae separately using the QIAGEN DNeasy Blood and Tissue Extraction Kit and used as a template in both conventional and qPCRs.

### 2.13. Detection of Stephanofilaria sp. DNA with BF Background

To ensure that the BF DNA did not interfere with the *Stephanofilaria* sp. DNA detection, mixtures of DNA were tested with qPCR. BF DNA was diluted 2-fold from 2400 ng/µL to 75 ng/µL, and each dilution was mixed with 1 pg/µL of *Stephanofilaria* sp. DNA and tested in qPCR assays. In addition, 1 pg/µL of *Stephanofilaria* sp. DNA and 2400 ng/ µL of BF DNA were run independently as positive and negative controls, respectively.

### 2.14. Preliminary Validation of Stephanofilaria sp. Specific Assays Using Bovine Skin Lesion Biopsies

The ability of all three molecular assays to detect *Stephanofilaria* sp. DNA in cattle skin was validated by testing cattle skin lesion biopsies preserved in ethanol in Section 2.1. DNA was extracted from the skin lesion biopsies using a QIAGEN DNeasy Blood and Tissue Extraction Kit and 100 ng of DNA was used as a template in both conventional and qPCR assays as described above. The findings of molecular assays were compared to the direct detection of *Stephanofilaria* sp. in bovine skin lesions.

## 3. Results

### 3.1. Isolation of Adult Nematodes and Microfilariae

Nematodes were recovered from the lesions near the medial canthus of the eyes, of eight hides that were screened using the saline recovery method. The number of nematodes recovered from individual lesions was variable from 1 to 8. Sediments from all nematode-positive samples were also positive for microfilariae, whereas two lesions negative for adult nematodes were positive for microfilariae. 

### 3.2. Morphological Description of Stephanofilaria sp.

**Figure 1:**
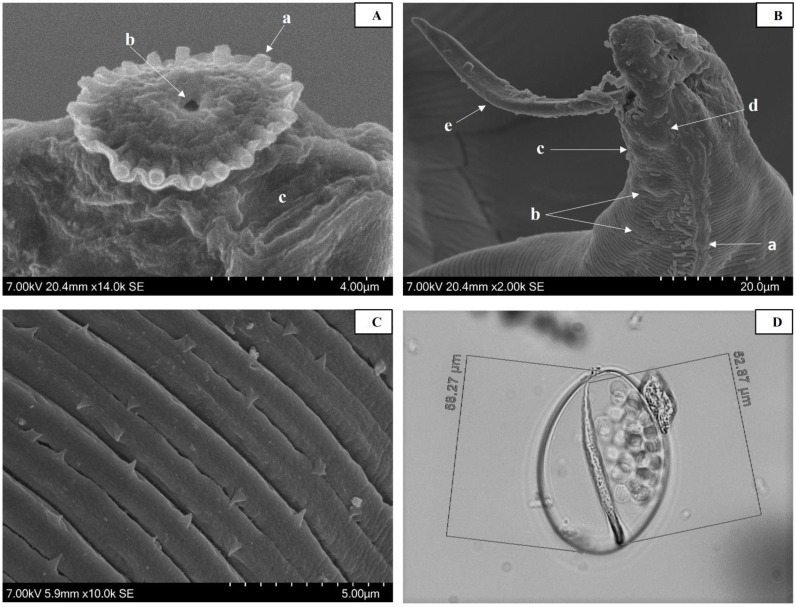
Morphology of *Stephanofilaria* sp.: (**A**) photomicrograph (SEM) of the anterior end of female adult *Stephanofilaria* sp. indicating peri-buccal spines (a), oral opening (b) and amphid (c); (**B**) photomicrograph (SEM) of the posterior end of adult male *Stephanofilaria* sp. indicating lateral ala (a), pre-anal papillae (b), median papillae (c), Ad-anal papillae (d), and left spicule (e); (**C**) photomicrograph (SEM) of the cuticle of adult *Stephanofilaria* sp. showing cuticular striations with spines; (**D**) photomicrograph (light microscopy) of microfilaria of *Stephanofilaria* sp. enclosed in a round to oval shape vitelline membrane with small spherical bodies.

Adults were small, slender, and creamy white in appearance. Males (*n* = 4) were 2.17–2.75 mm long, whereas females (*n* = 6) were somewhat larger but coiled and difficult to measure accurately. The anterior end of the adults had 18 distinct peri-buccal spines, a cephalic ridge without cephalic spines, one pair of large semi-circular amphids (Figure 1A), and one pair of large cephalic papillae. Males showed a large protrusible left spicule and small right spicule, with seven pairs of ventrolateral preanal papillae, a single median papilla, one pair of adanal, and one pair of postanal papillae at the caudal end (Figure 1B). The vulva in females was close to the anterior end with membrane-encapsulated microfilariae visible in the vagina and uterus of some specimens. Prominent raised lateral alae commenced on the anterior end, extending the full length of the body. Fine striations with anteriorly and posteriorly directed spines covered the cuticle of adults (1C), except on anterior and posterior ends. Microfilariae (*n* = 8) were 52.87–60.94 µm long, with a slightly wider anterior end, tapering posteriorly, and were enclosed in a round to oval-shaped vitelline membrane containing numerous small spherical bodies (Figure 1D).

### 3.3. Amplification, Sequence, and Phylogenetic Analysis of Stephanofilaria sp. cox1 mtDNA

**Figure 2:**
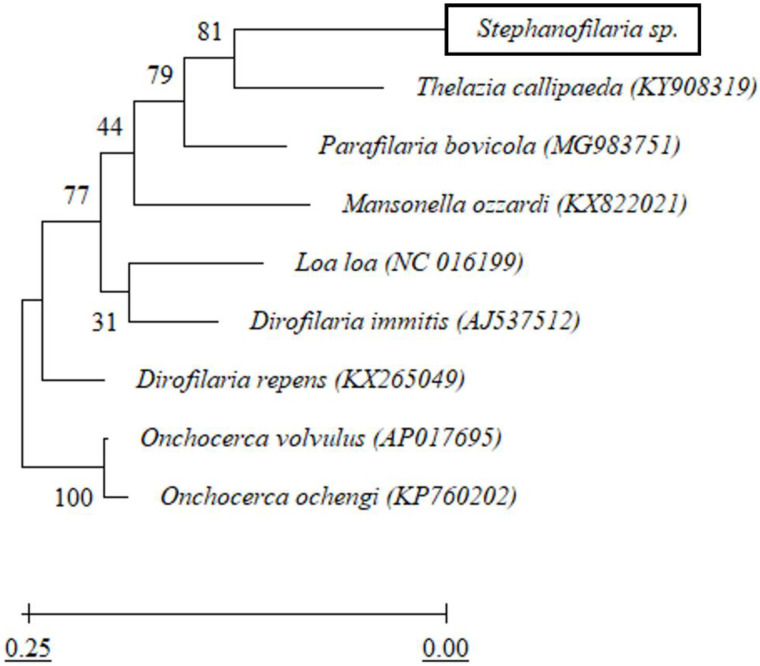
Maximum likelihood phylogenetic tree built from *cox1* nucleotide alignment of *Stephanofilaria* sp. and closely related filarial nematodes. Bootstrap branch support (based on likelihood analysis) is shown. Terminal branch labels provide source species names and GenBank accession numbers.

The PCR targeting the nematode conserved *cox1* gene resulted in a product of 730 bp, which after trimming of ends yielded a 606 bp fragment from two different *Stephanofilaria* DNA preparations from identical adults isolated from two different cattle lesions. Sequence comparison of the *Stephanofilaria* sp. *cox1* gene using BLASTn showed 85.28% identity with *D. repens* (accession no. KX265049)*,* 84.98% with *D. immitis* (accession no. AJ537512)*,* 84.41% with *T. callipaeda* (accession no. KY908319), and 84.26% with *O. volvulus* (accession no. AP017695). In all the above *cox1* sequences, query cover was 99–100%. A phylogenetic tree of the *cox1* alignment indicated that *Stephanofilaria* sp. is most closely related to *T. callipaeda* with bootstrap support of 81% (Figure 2). Both *Stephanofilaria* sp. and *T. callipaeda* were recovered sister to *P. bovicola* with the bootstrap support of 79%. The novel *Stephanofilaria* sp. *cox1* sequence was deposited into the GenBank database under accession no. MW143322.

### 3.4. Development and Optimisation of Stephanofilaria sp. Specific PCR Assays Based on ITS2 rDNA

#### 3.4.1. Amplification and Sequencing of the ITS2 rDNA Segment

The nematode conserved PCR targeting the rDNA segment resulted in a product of 889 bp which after trimming the ends yielded a 664 bp fragment. Two ITS2 sequences were obtained from two different *Stephanofilaria* DNA preparations from identical adults isolated from two different cattle lesions. BLASTn sequence comparison of the *Stephanofilaria* sp. rDNA segment showed that the query cover was between 36 and 40% associated with the 5′ region only of the 28S rDNA, the 404 bp ITS2 sequence alone had no matches in the GenBank database. The 28S rDNA region showed 86.6% identity with *T. callipaeda* (accession no. MF953480)*,* 83.82% with *O. gibsoni* (accession no. M15308)*,* 83.68% with *L. loa* (accession no. XR_002252420), and 83.56% with *D. immitis* (accession no. AF188120). The 28S rDNA sequence region was small and could not be aligned confidently and was thus not suitable for phylogenetic analysis. The rDNA sequence of *Stephanofilaria* sp. has been deposited into GenBank under accession no. MW143573. 

#### 3.4.2. Conventional PCR Development

*Stephanofilaria* sp. specific primers were designed based on the novel sequenced ITS2 region which yielded a product of 199 bp. The *Stephanofilaria* sp. specific PCR product was sequenced in both directions and was confirmed to specifically amplify the targeted ITS2 region. The conventional PCR successfully amplified DNA extracted from a single adult nematode.

#### 3.4.3. qPCR Assay Development

SYBR Green qPCR assays were performed using the same primer set as used in conventional PCR. The SYBR Green assay amplified the microfilariae DNA from the samples which were negative with conventional PCR, indicating a higher sensitivity than conventional PCR. For the SYBR green assay, melt curves were also analysed to confirm the presence of a single specific PCR product with a specific melting temperature of 76 °C for the *Stephanofilaria* sp. PCR product. The TaqMan qPCR specifically amplified a product of 76 bp from the ITS2 region and successfully detected both nematode and microfilariae DNA. 

#### 3.4.4. Specificity and Sensitivity Analysis

**Figure 3:**
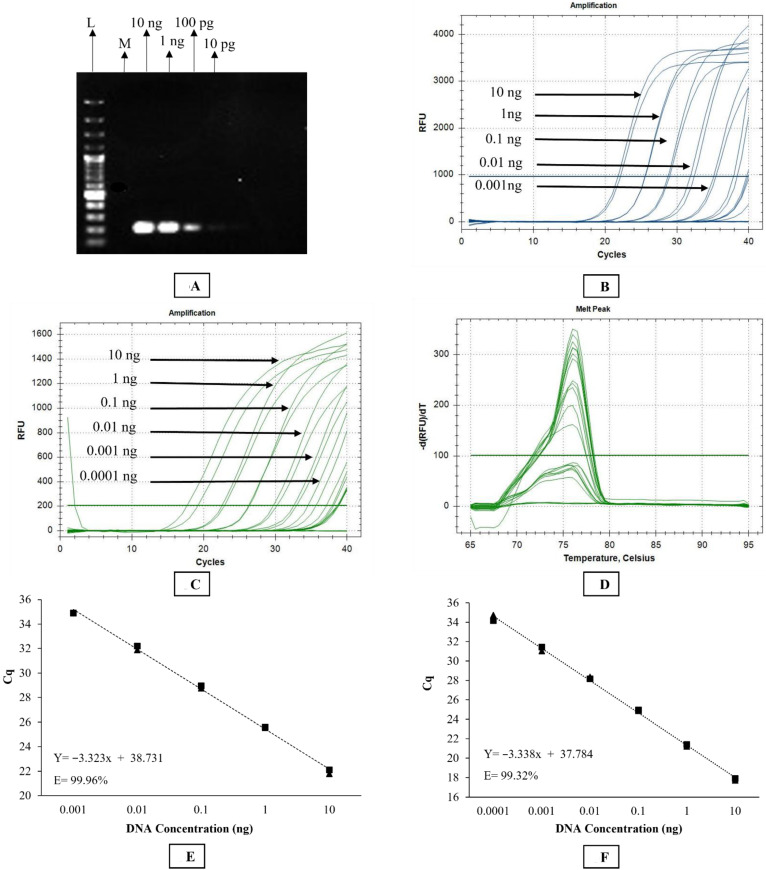
Sensitivity analysis of *Stephanofilaria* sp. specific PCR assays: (**A**) the sensitivity of conventional PCR assay was determined by visualisation of single specific PCR product band through agarose gel electrophoresis. Lane “L” and “M” indicate 100 bp DNA ladder and negative template control, respectively, while the concentrations of *Stephanofilaria* sp. DNA is shown above each lane with the estimated PCR product size 199 bp; (**B**) TaqMan qPCR amplification plot for *Stephanofilaria* sp. DNA dilutions along with DNA concentrations; (**C**) SYBR Green qPCR amplification plot for *Stephanofilaria* sp. DNA dilutions along with DNA concentrations; (**D**) melt curve for the SYBR Green qPCR; (**E**) standard curve of TaqMan assay for 10-fold serial dilutions of *Stephanofilaria* sp. DNA, indicating 99.96% efficiency with the slope of −3.323; (**F**) standard curve of SYBR Green assay for 10-fold serial dilutions of *Stephanofilaria* sp. DNA, indicating 99.32% efficiency with the slope of −3.338.

No amplification was observed with *O. gibsoni* or *D. immitis* DNA using any of the *Stephanofilaria* sp. primers. Similarly, there was no background amplification observed from BF or bovine skin DNA with either conventional or qPCR assays. Conventional PCR detected down to 1 ng of *Stephanofilaria* sp. DNA (Figure 3A). The sensitivity of *Stephanofilaria* sp. DNA amplification was 1 pg (Cq = 35.28) and 100 fg (Cq = 33.6) for TaqMan and SYBR green assays, respectively (Figure 3B–D). The Cq values obtained from both qPCR assays were inversely proportional to the amount of DNA template tested, and the reaction efficiencies for TaqMan and SYBR Green assays were 99.96% and 99.32%, respectively (Figure 3E,F). 

#### 3.4.5. Validation of Assays to Detect Microfilariae DNA

**Figure 4:**
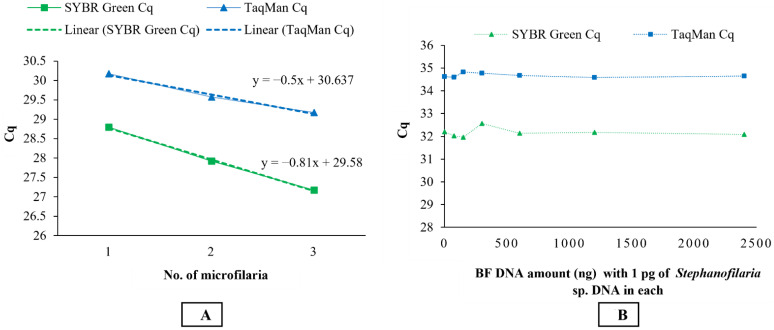
(**A**) Graph showing the inverse relationship between the number of microfilariae and Cq value, with slopes of −0.5 and −0.81 for TaqMan and SYBR Green qPCR, respectively; (**B**) graph explaining no significant change in Cq value of TaqMan and SYBR Green qPCR as compared to the positive control. Zero value on the x-axis indicates positive control (1 pg of *Stephanofilaria* sp. DNA). One pg of *Stephanofilaria* sp. DNA was detected with the same Cq value in all BF DNA concentrations.

Microfilariae DNA samples were negative using conventional PCR. The qPCR assays detected DNA from single, two, and three microfilariae with the TaqMan^TM^ Cq values of 30.17, 29.57, and 29.17, respectively, while the SYBR Green assay recorded Cq values of 28.79, 27.92, and 27.17 from single, two, and three microfilariae DNA templates, respectively. An inverse relationship between the number of microfilariae and Cq values with the slope of −0.5 and −0.81 was observed for TaqMan and SYBR Green assays, respectively (Figure 4A). 

#### 3.4.6. Detection of Microfilariae DNA with BF Background

All BF DNA dilutions spiked with 1 pg/µL *Stephanofilaria* sp. DNAs were detected at the same Cq value as that of the positive control (1 pg *Stephanofilaria* sp. DNA), indicating no interference of BF DNA in nematode detection. The Cq’s for all positive samples from 31.96–32.56 and 34.58–34.82 with SYBR Green and TaqMan assays, respectively (Figure 4B). 

#### 3.4.7. Preliminary Validation of *Stephanofilaria* sp. Specific Assays Using Bovine Skin Lesion Biopsies

The *Stephanofilaria* sp. specific molecular assays were initially validated by comparing both conventional and qPCR results for skin biopsies from 25 cattle with the findings of the saline recovery technique (Table 3). Conventional PCR detected 12 samples (44%) and both qPCRs detected 14 samples (56%) positive for *Stephanofilaria* sp. DNA. All samples in which nematodes or microfilaria were found by saline recovery were also determined as positive by both qPCR methods (Table 3). The overall sensitivity of saline recovery for *Stephanofilaria* sp. detection in these skin biopsies relative to the molecular assays was 71.4%.

## 4. Discussion

In Australia, BF-associated lesions have been speculatively associated with *Stephanofilaria* sp. nematode infection. However, the exact aetiology of these lesions is still unclear. In the USA, hypersensitivity induced by horn-fly feeding and the involvement of bacteria, in particular *Staphylococcus aureus*, have been suggested as contributing factors in the development of horn-fly associated lesions [28,29]. However, due to the lower sensitivity of available techniques for detecting *Stephanofilaria* sp. nematodes, investigating the pathogenesis and epidemiology of these lesions has been difficult. Therefore, to clarify the role of *Stephanofilaria* sp. in the development and epidemiology of BF lesions, a more sensitive detection technique was required. This paper reports the first conventional and qPCR assays to detect different life stages of *Stephanofilaria* in vector fly and definitive hosts, as well as preliminary morphological and phylogenetic analyses for this novel nematode species. 

In this study, the nematodes were isolated from lesions near the medial canthus of eyes from cattle and from the same area of Queensland from which Johnson [6] first identified *Stephanofilaria* sp. in Australia. The morphology of the nematodes, geographical location, and the clinical appearance of lesions from which the nematodes were collected, as well as their detection in buffalo flies, indicates that the nematodes were similar to those described previously [6]. The two most closely related genera of filarial nematodes described from cattle with similar cutaneous locations are *Onchocerca* spp. and *Parafilaria* spp. In Australia, *Onchocerca* spp. are transmitted by *Culicoides *marksi*,* *Forcipomyia (Lasiohelea) townsvillensis,* and *Austrosimulium pestilens* [30] and cause cutaneous nodules in cattle. The adult *Onchocerca* species are much larger (up to 50 cm) than the nematodes examined in this study [8]. *Parafilaria bovicola* are mainly transmitted by *Musca* spp., and the adults are of similar in size to *Stephanofilaria*, but they have not to date been reported from Australian cattle [8]. In addition, gravid female nematodes isolated from the skin lesions in this study carried microfilariae enclosed in spherical vitelline membranes within the uterus, and similar membrane-enclosed microfilariae were isolated from host tissues. This is consistent with previous morphological studies of microfilariae from other species within the genus *Stephanofilaria* [6,7,31,32]. In comparison, *Onchocerca* spp. and *Parafilaria* spp. have unsheathed microfilariae [24,30].

Scanning electron micrographs also suggest that the nematodes in this study are the same species as those reported in the past [6]. The only differences in the morphological features described in this study were in the number of peri-buccal spines observed and the presence of cuticular spines. Johnson [6], using light microscopy, observed 15–16 peri-buccal spines, whereas 18 were counted using SEM in our study. In addition, Johnson [6] reported no cuticular spines, while cuticles with spines directed both anteriorly and posteriorly were observed in this study. It is difficult to count the exact number of peri-buccal spines using light microscopy, and Johnson [6] noted that counting of peri-buccal spines was imprecise, as the spines were difficult to distinguish from the supporting collar. This difference is relatively minor, and the higher number of peri-buccal spines noted here is likely the result of the clearer definition possible using SEM. In common with previous observations, no cephalic spines were observed which suggests that this characteristic may differentiate the Australian *Stephanofilaria* sp. [6] from *S. stilesi* vectored by *H. irritans* which was reported to have 4–5 cephalic spines [7]. Further determination of the species status of the Australian *Stephanofilaria* and *S. stilesi* will require a more detailed morphological and molecular comparison of the two morphotypes. 

Mitochondrial DNA is genetically conserved within species in the same genus, and *cox1* sequences have thus been used previously in many studies to reveal phylogenetic relationships among species [24,33,34,35,36,37]. In addition, rDNA (ITS1 and ITS2) has been previously used for delineation and identification of filarial nematodes [17,38]. Thus, amplification and sequencing of the *cox1* gene were used for the determination of the phylogenetic relationship with other representing species closely related to *Stephanofilaria* sp. The ITS2 region was chosen as a suitable target for use in specific diagnostic assays for *Stephanofilaria* sp. Internal transcribed spacer (ITS)-based PCR assays have already been reported for specific detection of different nematodes from order Strongylida, Ascaridida, and Spirurida [16,18,19,20,21,22,23,24].

From the BLASTn sequence comparison, the *cox1* and partial rDNA sequences of *Stephanofilaria* sp. showed maximum similarities to *Dirofilaria*, *Onchocerca*, *Thelazia,* and *Loa* spp. At first glance, this was not surprising, as the genus *Stephanofilaria* is classified within the superfamily Filarioidea [39]. Among the most similar genera from this group, *Dirofilaria* and *Onchocerca* belong to the family Onchocercidae, but *Thelazia* belongs to the family Thelaziidae. However, the genus *Stephanofilaria* is currently placed in the family Filariidae. For the Filariidae, *cox1* and rDNA sequences hitherto have been available in GenBank for species of *Parafilaria*, *Brugia,* and *Loa.* Of these, members of the genus *Loa* showed the highest similarity (83.68%) with *Stephanofilaria* sp. Overall, no other member of the family Filariidae was among the top 20 BLASTn hits. Phylogenetic analysis in this study indicated that *Stephanofilaria* sp. shares a closer common ancestor with *T. callipaeda* than with any member of the family Filariidae. This close association is of particular interest given the similar biology and close proximity of infestations of *T. gulosa* and *T. skrjabini* in Australian cattle [40]. The *Stephanofilaria* sp. nematodes used in this study were collected from lesions on the medial canthus of the eye, whereas *Thelazia* spp., previously isolated from Australian cattle, were found in the conjunctival sac, under the third eyelid and within the lachrymal ducts and nasolachrymal canal [40]. Interestingly, *Stephanofilaria* has been moved between families in the order Spirurida on several occasions based on its unique morphological and biological characteristics and the predilection site of the nematode [41]. Boomker et al. [2] isolated *Stephanofilaria* nematodes from the skin of a hippopotamus which he named *Stephanofilaria thelazioides* n. sp because of morphological similarities to the genus *Thelazia*. The results of this study may suggest that *Stephanofilaria* should be placed in the family Thelaziidae but more conserved *Stephanofilaria* genes, and more sequences from other *Stephanofilaria* species and their close relatives are required to support this claim.

The rDNA sequence from our study contained the complete ITS2 and partial 28S segment. The ITS2 sequence of *Stephanofilaria* sp. had no similarity with any sequence available in GenBank. Due to the greater divergence in the ITS2, it was used to design species-specific conventional and qPCR assays for the identification and delineation of both adult and larval stages of *Stephanofilaria* sp. Both conventional and qPCR assays specifically amplified *Stephanofilaria* sp. DNA with no amplification for other filarial nematode DNA tested in these studies. Comparison of the Cq values from microfilariae DNA to the standard curve for SYBR Green and TaqMan qPCR indicated that individual microfilaria yields a low amount of DNA (150–200 pg) and the lower number of cycles we used for the conventional PCR (30 cycles), compared to qPCR assays (40 cycles), significantly affected the assay sensitivity. Quantitative PCR assays are highly sensitive and relatively advanced tools for high-throughput detection of target DNA and are rapidly replacing conventional PCR and microscopic methods as detection methods for parasites. Both the SYBR Green and TaqMan assays developed in this study were able to detect DNA from individual microfilaria with a reliable Cq (<31). Overall, the SYBR Green assay appeared to be 10 times more sensitive than the TaqMan assay, and this has also been observed in the detection of *Babesia* spp. [42]. However, melting temperature analysis is needed to ensure specificity of the SYBR Green assay and this could be difficult under some circumstances. This suggests that the TaqMan assay may be more suitable for general use to ensure cleaner results and more robust diagnostic specificity. 

The significantly higher sensitivity of PCR-based assays for nematode detection compared to traditional histological methods has been reported in previous studies with other nematode species [16,43,44]. Similarly, our findings showed that molecular assays were more sensitive than saline recovery detection. Although the qPCR technique detected as little as 100 fg of *Stephanofilaria* sp. DNA, the sensitivity of detection of *Stephanofilaria* sp. in the skin lesions, will also be strongly dependent on the sampling methods used. The higher sensitivity of the qPCR assays may also allow detection from non-invasive samples (e.g., from lesion swabs), as there is evidence of the presence of skin infecting nematodes and microfilariae at the surface of active lesions [24]. Quantitative PCR assays detected nematode DNA in four skin samples which were negative for the presence of nematode or microfilariae using the saline recovery technique. However, it is yet to be confirmed whether molecular detection relies on the presence of whole or partial nematodes or microfilariae in the sample collected, or whether the PCR may be able to detect the presence of *Stephanofilaria* sp. from secretory or excretory compounds or cellular material abraded during nematode migration. 

This study also showed that there was no effect on the sensitivity of qPCR assays in detecting 1 pg of *Stephanofilaria* sp. DNA with high background concentrations (2400 ng/ µL) of buffalo fly DNA. An amount of 1 pg is approximately 0.5–0.66% of DNA found in single microfilaria, suggesting that both SYBR Green and TaqMan assays could be used to detect first-, second-, and third-stage *Stephanofilaria* sp. larvae in BF. Quantitative PCR-based detection techniques have also been used to detect *Brugia malayi* and *Wuchereria bancrofti* in mosquitoes [45,46]. The PCR-based assays developed here would overcome the need for time-consuming dissection and examination of BF to determine the presence of infection. Furthermore, these assays may also have application for use in epidemiological studies with other species of *Stephanofilaria* in other vector and host associations, especially closely related *S. stilesi*, but this requires confirmation.

## 5. Conclusions 

SYBR Green and TaqMan PCR assays developed in this study specifically detected *Stephanofilaria* sp. both in vector and host regardless of developmental stage. Accurate detection and identification of *Stephanofilaria* sp. will help to determine its distribution and epidemiology, and assist in determining the aetiology of skin lesions towards the development of optimal methods for treatment and control. In addition, SEM and phylogenetic information from this study will help clarify the systematic status of *Stephanofilaria*, although further studies will be required to this end. 

## Figures and Tables

**Table 1:** Primers and probes for the Polymerase chain reaction assays for *Stephanofilaria* sp.

Assay	Primer Name	Sequences	Tm °C	GC%	Product Size in Base Pairs
*cox1*	St_CO1F	ATACTGTKAATCATAAGACTATTGG	65	32	700 bp
	St_CO1R	GACCAAAAAATCAAAACAAATGCTG	66	30.7	
rDNA	S_5.8S_29F	TAGCGGTGGATCACTTGGCTCG	59	59.1	900 bp
	28S_400R	CAACTTTCCCTCACGGTACTTGT	55	47.8	
*Stephanofilaria* sp. ITS2	S_ITS2_F2	GGCAATAGTATGCATATAATAAATGTG	60.3	29.6	199 bp
	S_ITS2_R2	TTCAGCGGGTAATCTCGACT	63.7	50	
TaqMan (ID: AP326KJ)	AP326KJ_F	CAGCAATGTTCATAATGTGACCTCAA			76 bp
	AP326KJ_R	TTTCCTCCGCTTAGTGATATGCTTA			
	AP326KJ_Probe	FAM-CAGCGGGTAATCTC			

**Table 2:** List of all the nematodes sequences and their corresponding accession numbers used in this study for phylogenetic analysis.

Species Name	Accession Number
*Onchocerca volvulus*	AP017695
*O. ochengi*	KP760202
*Dirofilaria immitis*	AJ537512
*Dirofilaria repens*	KX265049
*Mansonella ozzardi*	KX822021
*Loa loa*	NC016199
*Parafilaria bovicola*	MG983751
*Thelazia callipaeda*	KY908319
*Stephanofilaria* sp. (submitted from this study)	MW143322

**Table 3:** Comparison of the results of saline recovery technique and *Stephanofilaria* sp. specific molecular assays. Positive PCR results are indicated by +, and negative results by −.

Collection Date	Cow ID	Saline Recovery Technique	Molecular Assays		
			Conventional	TaqMan	SYBR Green
May 2019	C-1	−	−	−	−
	C-2	N & MF	+	+	+
	C-3	−	+	+	+
	C-4	−	−	+	+
	C-5	−	−	−	−
	C-6	−	−	−	−
	C-7	−	−	−	−
	C-8	−	−	−	−
	C-9	−	−	−	−
	C-10	−	−	−	−
September 2019	C-11	−	−	−	−
	C-12	−	−	−	−
	C-13	−	−	−	−
	C-14	MF	+	+	+
	C-15	−	−	+	+
	C-16	−	−	−	−
	C-17	N & MF	+	+	+
	C-18	N & MF	+	+	+
	C-19	N & MF	+	+	+
	C-20	MF	+	+	+
	C-21	N & MF	+	+	+
	C-22	N & MF	+	+	+
	C-23	N & MF	+	+	+
	C-24	N & MF	+	+	+
	C-25	−	+	+	+
Total positives		10	12	14	14

N: adult nematode; MF: microfilariae.

## Data Availability

The novel *cox1* and ITS2 sequences of *Stephanofilaria* sp. were deposited into the GenBank database under accession no. MW143322 and MW143573, respectively. The data will be publicly available when the article citing these accession numbers is published but are available from the corresponding author on reasonable request.
